# ILC2 Activation by Protozoan Commensal Microbes

**DOI:** 10.3390/ijms20194865

**Published:** 2019-09-30

**Authors:** Kyle Burrows, Louis Ngai, Flora Wong, David Won, Arthur Mortha

**Affiliations:** 1 University of Toronto, Department of Immunology, Toronto, ON M5S 1A8, Canada; kyle.burrows@utoronto.ca (K.B.) louis.ngai@mail.utoronto.ca (L.N.); fl.wong@mail.utoronto.ca (F.W.); taehyoan.won@mail.utoronto.ca (D.W.); 2 Ranomics, Inc. Toronto, ON M5G 1X5, Canada

**Keywords:** ILC2, protozoa, Trichomonas, *Tritrichomonas musculis*, mucosal immunity, taste receptors, succinate, intestinal immunity, type 2 immunity, commensals

## Abstract

Group 2 innate lymphoid cells (ILC2s) are a member of the ILC family and are involved in protective and pathogenic type 2 responses. Recent research has highlighted their involvement in modulating tissue and immune homeostasis during health and disease and has uncovered critical signaling circuits. While interactions of ILC2s with the bacterial microbiome are rather sparse, other microbial members of our microbiome, including helminths and protozoans, reveal new and exciting mechanisms of tissue regulation by ILC2s. Here we summarize the current field on ILC2 activation by the tissue and immune environment and highlight particularly new intriguing pathways of ILC2 regulation by protozoan commensals in the intestinal tract.

## 1. The ILC Lineage

### 1.1. The Family of Innate Lymphoid Cells

Research over the last decade has redirected focus away from classical immune cell interactions within lymphoid tissues towards immunity within non-lymphoid tissues. Within these tissues, immune interactions involve local adaptation and rapid responses by tissue-resident immune cells. While seminal work on tissue-resident macrophages proposed locally produced, tissue-specific signals as regulators of immune cell identity, these cues still remain less well-defined for lymphoid cells [1]. Lymphoid growth factors like interleukin(IL)-7, IL-15, or thymic stromal lymphopoietin (TSLP) are critical for the maintenance of tissue-resident memory T cells and plasma cells that seed tissues and provide antigen-specific protection after an immune response [2]. Interestingly, the identification of tissue-resident lymphoid cells lacking B and T cell markers while retaining the expression of common-gamma chain (CD132)-associated receptors for IL-7, IL-15, or TSLP (i.e., CD127/IL-7Ra, CD122/IL-2Rb, and cytokine receptor-like factor/Crlf2/TSLPR) revealed the presence of innate lymphoid cells (ILCs) in almost every solid organ [3]. These lymphocytes rapidly secrete cytokines upon stimulation and facilitate early/immediate responses against local changes in the tissue environment [4,5]. At least four different groups of ILCs can now be discriminated with tissue-dependent homing preferences based on distinct homing receptor profiles [6]. These four groups, include natural killer (NK) cells, group 1 ILCs (ILC1), group 2 ILCs (ILC2), and group 3 ILCs (ILC3), provide type 1, type 2, and type 3 immune responses through their respective effector function and play an important role in orchestrating antimicrobial immunity against viruses, intracellular and extracellular microbes, and parasites [7].

The commitment towards the ILC lineages happens at the early innate lymphoid progenitor (EILP) stage, while diversification of ILCs, dictated by lineage-defining transcription factors, appears in the common innate lymphocyte progenitor (CILP) that restricts their potential to only generate group 1-3 ILCs and NK cells. Though NK cells are considered part of the ILC family, they differ from conventional ILCs, as they are capable of killing target cells (similar to CD8+ T cells) [8]. Interestingly, NK cells and their respective precursor were shown to diverge from the ILC1, 2, and 3 lineages prior to reaching the common helper innate lymphocyte precursor (CHILP) stage [9]. Instead of executing cell killing, ILC1-3s arising from CHILPs are major producers of cytokines that mirror classical CD4 helper T cells (Th1, Th2, and Th17 respectively) in most of their functional features [10]. The ability of ILCs to rapidly respond to locally produced cytokines establishes them as key elements that integrate tissue-specific signals into effector functions. These functional features make ILCs an interesting type of immune cell, capable of modulating the local/tissue-specific cytokine milieu for newly infiltrating immune cells. Environmental factors like nutrients, xenobiotics, or the microbiome are additional factors, besides pathogens, that play an important role in shaping the function and microenvironment of mucosal organs [11]. We have previously discussed the interactions and control-circuits of ILCs and myeloid cells at these mucosal surfaces and will instead focus our attention on summarizing currently known tissue-specific entities including the microbiome and their role in regulating local type 2 immunity by ILC2s. We will close this review by focusing on particularly new, emerging microbial members of the intestinal microbiome and their implication in regulating ILC2 functions [7].

### 1.2. Transcriptional Specification of the ILC2 Lineage

**Figure 1:**
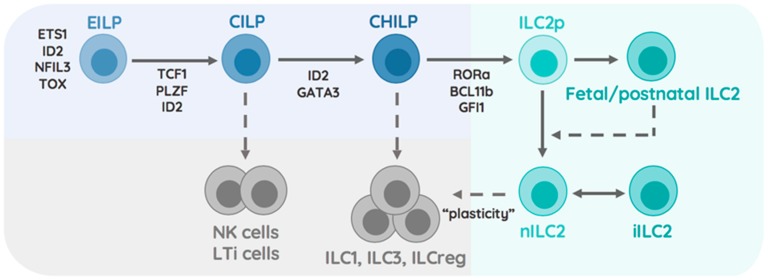
Development of ILC2. This scheme demonstrates the subdivision of the ILC2 lineage into oligo-potent precursors (Blue), non-ILC2 lineages (Grey), and the ILC2 lineage (Green). The Early Innate Lymphocyte Precursor (EILP) expresses ETS1, ID2, NFIL3, TOX, and differentiates into the Common Innate Lymphocyte Precursor through the expression of TCF1 and PLZF. These precursors give rise to NK cells, LTi cells (dashed arrow) and the Common Helper Innate Lymphocyte Precursor (CHILP). ID2 and GATA3-dependent CHILP lose the potential to generate NK cells and LTi cells while retaining the ability to generate ILC1, ILC3, and ILCreg (dashed arrow). Upregulation of RORa, BCL11b, and GFI1 within CHILP restrict the development of ILC2 progenitors that differentiate into fetal/postnatal ILC2 and adult/natural ILC2. Fetal/postnatal ILC2 may differentiate into natural ILC2 (dashed arrow). Natural ILC2s differentiate into inflammatory ILC2 under circumstance of tissue perturbation and respond to environmentally-induced signal by destabilizing their ILC2 phenotype and adapting ILC1/ILC3 effector functions possibly termed ‘plasticity’ (dashed arrow).

ILC2 arise from CHILPs via differentiation from ILC2 precursors (ILC2p) that can be found in both the fetal liver and adult bone marrow [12]. Differentiation of ILC2p into immature ILC2 and mature ILC2 is mediated by upstream cytokines such as IL-25, IL-33, and TSLP secreted by epithelial cells and myeloid cells [13]. These cytokines, alongside lipid mediators and receptor-ligand interactions, initiate the expression of transcription factors that regulate the differentiation pathway into mature ILC2. The transcription factor retinoic acid-related orphan receptor alpha (RORa) was found to be a critical driver of ILC2 development in mice carrying the spontaneous *RORa-sg/sg* mutation [14,15]. Zinc finger protein 163, also called growth factor independent 1 transcriptional repressor (encoding Gfi1), was shown to control ILC2 development and function through regulation of the transcription factor GATA binding protein 3 (GATA3) [16]. Follow-up studies further demonstrated the dependency of ILC2 development on GATA3 by conditionally deleting *Gata3* in all helper like ILCs via inducible expression of Cre recombinase in all *Inhibitor of DNA binding 2* (*Id2*)-expressing cells, which resulted in a lower frequency but not complete absence of ILC2 [12]. Interestingly, GATA3 expression is controlled by Notch activation through delta-like ligands in T lymphocytes and promotes their differentiation into Th2 cells [17]. In line with these reports, mice lacking the transcription factor T cell factor 1 (Tcf1, encoded by *Tcf7*), a major downstream target of Notch, were found to show a significant impairment in GATA3-dependent ILC2 development [18,19]. Interestingly, ILC3 also express low levels of GATA3 and require Notch activation for further differentiation into natural cytotoxicity receptor (NCR)-expressing ILC3 [19]. Similar to Tcf1, another gene acting as early T cell-specifying regulator is B-Cell Lymphoma/Leukaemia 11B (encoded by *Bcl11b*) [20]. Surprisingly, only one-third of all early ILC precursors were found to express Bcl11b and conclusively, mice deficient in Bcl11b demonstrated a severe stall in their ability to differentiate into ILC2s [21]. Following commitment to the ILC2 lineage, stability of this lineage through repression of type 3 signature genes is ensured by the transcription factor Gif1 and the lysine methyltransferase G9a [15,16,22,23]. Collectively, these findings demonstrate the importance of GATA3, RORa, Tcf-1, Bcl11b, Gif1, and histone-modifications as critical elements in the ILC2 lineage (Figure 1).

### 1.3. ILC2 Heterogeneity Across Tissues

The initial characterization of ILC2s as Gata3^hi^ killer cell lectin-like receptor subfamily G member 1 (KLRG1) expressing cells was soon expanded with high-dimensional single cell RNA-sequencing, uncovering the full diversity of tissue-resident ILC2s. The analysis of intestinal ILCs on the single cell level revealed the presence of at least four distinguishable ILC2 subsets within the intestinal tract [24]. More strikingly, single cell analysis of ILC2s across the bone marrow, fat, skin, lung, and intestinal tract demonstrated that tissue-derived signals were determinants of ILC2 identity and transcriptional adaptation [25]. Distinct subsets across tissues were found to preferentially respond to locally produced environmental cytokines, particularly highlighting the presence of an IL-18R expressing ILC2 in the skin that contributed to the development of atopic dermatitis [25]. Both reports identified Arg1-expressing ILC2 within the intestinal tract, showing particular enrichment of these cells within the adipose and lung tissue [24,25]. Considering that ILC2s are capable of proliferating within organs and execute interorgan trafficking in an S1P-dependent manner in the presence of inflammation, it raises questions about the stability of these subsets within individual tissue [26]. Fate-labelling studies of ILC2s during pre- and postnatal periods identified that neonatal ILC2 seed developing tissues early during life and contribute to the pool of ILC2s found in adult mice (Figure 1). Characterizing gene expression in adult and neonatal ILC2s associated the production of IL-5 and IL-13 with neonatal ILC2s [27]. These findings highlight that ILC2 subsets are distinct, not only by anatomic location, but also by ontogenetic time of birth. These findings should inspire research aiming to understand how early life perturbations of tissues or immune cells affects ILC2 heterogeneity and infection outcomes.

## 2. Activation and Inhibition of ILC2

### 2.1. Cytokine Receptor Profiles

ILC2s are largely tissue-resident cells, adopting phenotypes dictated by their local tissue environment [28]. However, considering that ILC2s have a substantial degree of plasticity and are able to adopt an ILC1 or ILC3-like phenotype, they have also been found to leave their resident tissues via lymphatic vessels [29,30]. Signaling pathways by cytokine receptors on ILC2s are critical to induce these functions and show differential expression on ILC2s in a tissue-dependent manner, collectively suggesting a coordinated cytokine receptor profile to anticipate distinct tissue environmental signals [25].

**Figure 2:**
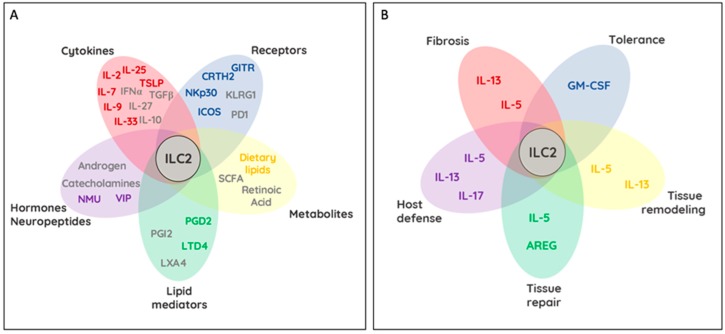
Regulation of ILC2 functions and ILC2-dependent control of host tissues. Schemes demonstrate in (**A**) molecules and receptors involved in ILC2 activation (colored) and ILC2 inhibition (grey). Bubbles within the Venn-diagram like schemes represent subclasses of activating and inhibiting molecules (Cytokines—red; non-cytokine, surface receptor—blue; dietary metabolites— yellow; lipid mediators—green; neuropeptides and hormones—violet). Overlaying bubbles indicate possible synergistic and antagonistic actions of these molecules that in sum positively and negatively regulate ILC2 activation. Scheme (**B**) demonstrates the effects of ILC2 activation on tissues and immunity. Selective cytokines and cytokine groups are indicated. Tissue remodeling, tissue repair, host defense, fibrosis, as well as immune and disease tolerance require selective and context dependent actions of IL-5, IL-13, IL-17, AREG, and GM-CSF. Overlap between individual bubbles within the Venn-diagram further suggest possible transitions between tissue-regulatory effects by ILC2.

ILC2s are identified by the absence of lineage markers, and distinguished from other ILCs by the combined expression of IL-2Ra (CD25), IL-4Ra (CD124), IL-7R (CD127), IL-9R (CD129), IL-25R (IL-17Ra and IL-17Rb), IL-33R (ST2 and IL-1RAcP), TSLPR, DR3 (TNFRSF25), and c-kit (CD117) [13] (Figure 2A). These cytokine receptors trigger activation, survival and proliferation of ILC2s and share overlapping and unique signaling modules. For example, in vitro cultures of ILC2s demonstrated a pro-survival effect when cultured in the presence of TSLP, while stimulation via IL-33 alone, or in combination with IL-2, IL-25, or TSLP led to an increase in cytokine production [31]. Interestingly, stimulation with different combinations of cytokine changed the expression pattern of cytokine receptors likely rendering ILC2 permissible to cytokines produced by different cellular sources [31]. IL-2 appeared to additionally boost ILC2 activation by further amplifying the production of IL-5, IL-13, and GM-CSF [31].

The sources of these cytokines are varied and include T helper cells, basophils, stem cells, mast cells, tuft cells, epithelial cells, and macrophages [7]. Interestingly, IL-9 produced by ILC2s led to the activation and increased release of IL-5 and IL-13 through an autocrine loop via the ILC2-expressed IL-9R [32]. This loop further had a beneficial impact on the survival of ILC2 by upregulating Bcl-3, suggesting a possible loop for self-maintenance via IL-9 [33]. ILC2s are also targets of inhibitory signals. Inducible regulatory T cells (Tregs) negatively regulated ILC2s through secretions of IL-10 and TGF-β [34]. However, whether regulatory ILCs, sharing a highly overlapping cytokine profile with Tregs, are capable of inhibiting ILC2 functions via IL-10 and TGF-β remains an open question [35]. During type 1 immune responses like viral infections, type I and III interferons as well as IL-12 and IL-27 were found to downregulate cytokine production of ILC2s in a STAT1/STAT4-dependent fashion [29,36,37]. Intriguingly, the inflamed lung microenvironment, rich in the pro-inflammatory cytokines IL-1β and TSLP, primed ILC2s to produce IFN-γ in response to IL-12, with antagonizing function executed by IL-4 [38]. Collectively, these results demonstrate a profound degree of adaptation of ILC2s to changing tissue environments and open exciting possibilities to utilize this knowledge for tissue- and context-dependent control of ILC2 function during health and disease (Figure 2A). Additional mechanisms of ILC2 activation and communication exist, including cell-to-cell interactions with other tissue-resident hematopoietic and non-hematopoietic cells. Receptor ligand interactions between ILC2s and environmental cells have additionally been reported to regulate ILC2 activity and effector functions, depending on their cytoplasmic signaling motifs.

### 2.2. Activating and Inhibiting Surface Receptors on ILC2

#### 2.2.1. GITR (TNFRSF18)

The glucocorticoid-induced tumor necrosis factor receptor-related protein (*GITR*) is part of the TNF receptor superfamily and encodes a type 1 membrane receptor. GITR and its cognate ligand, GITRL, is known to be important in the regulation of inflammation and the immune response [39]. Its function is best characterized in T cells, where it plays a role in T cell survival [40,41]. GITR has also been found to be expressed by other immune cells including ILC2s, where it acts as a stimulatory receptor in the context of lung inflammation (Figure 2). Nagashima et al. compared *GITR*^−/−^ and WT mice under conditions of papain-induced lung inflammation and reported markedly decreased ILC2 numbers in the lungs accompanied by increased apoptosis and lower IL-5 and IL-13 production as a result of abrogated IL-9 release. These results suggest that GITR acts as a crucial driver of lung inflammation through the activation of ILC2s [42]. In contrast, GITR expression on IL-33 activated ILC2s may also be protective in the context of type 2 diabetes [43] (Figure 2). Here the researchers demonstrate that GITR engagement protected from both the onset and established insulin resistance through IL-13 secretion, which in the visceral adipose tissue, controlled the anti-inflammatory profile of alternatively activated macrophages [44]. Interestingly, islet-resident ILC2s were found to promote anti-diabetic effects through the induction of immune-regulatory functions in myeloid cells, which in turn favored the generation of Tregs within the pancreas [45]. These results show that GITR on ILC2s play a dual role in a disease-dependent context. More importantly, these findings suggest that appropriate ILC2 activation, survival, and cytokine production can also be achieved through GITR mediated activation of the NF-kB signaling pathway.

#### 2.2.2. CRTH2

Chemoattractant receptor homologous molecule expressed on Th2 cells Prostaglandin D2, mainly produced by activated mast cells, initiates signaling by binding to the G-protein coupled receptors PTGDR and CRTH2. CRTH2 is well characterized as a potent activator of Th2 cells and eosinophils [46]. CRTH2 has been found to be expressed on both mouse and human ILC2s [47] (Figure 2A). Similar to its role on Th2 cells and eosinophils, CRTH2 on ILC2s was shown to be important in their accumulation in the lung upon parasitic challenge [48] (Figure 2B). Similar dependency of ILC2 on CRTH2 signaling was reported for human ILC2s that upregulated ST2 and IL-17RA in response to PGD2 stimulation. Conclusively, stimulation of ILC2 with PGD2 in combination with IL-25 and IL-33 promoted elevated ILC2 cytokine responses [49]. In parallel to the IL-9 mediated autocrine stimulation of mouse ILC2, human ILC2s produced endogenous PGD2, which contributed to ILC2 activation in an autocrine fashion [50]. CRTH2 antagonists used in clinical trials demonstrated improvement of asthma and allergic rhinitis likely due to inhibitory effects on Th2 cells and ILC2 [47,51].

#### 2.2.3. ICOS/ICOSL (CD278)

Inducible T cell co-stimulator (ICOS) is member of the CD28 family, a molecule important in T cell signal transduction [52]. ILC2s co-express ICOS and its ligand, ICOSL, which aids in cell survival and effector function through homotypic interactions (Figure 2). The loss of ICOS on murine ILC2s or blocking of the ICOS:ICOSL interactions in human ILC2s reduced airway hyperreactivity and lung inflammation by interfering with cytokine-dependent STAT5 activation and resulting impairment in IL-13 release [53,54]. Moreover, the expression of ICOS on the surface of ILC2s further implicates it in the interactions between ILC2 and DCs, specifically through ICOS:ICOSL, which led to ILC2 activation [55]. Interestingly, development of idiopathic pulmonary fibrosis was inhibited through ICOS-engagement on IL-5 producing ILC2s, illustrating protective effects of ILC2 activation in the context of disease [56]. More recently, IL-33 mediated recruitment of Tregs was shown to suppress ILC2 activation via ICOS:ICOSL interactions. This mode of suppression was documented across different tissues and susceptible to inhibition by IFN-γ [34,57].

#### 2.2.4. KLRG1

The inhibitory killer cell lectin-like receptor G1 (KLRG1) is expressed by subsets of T cells and NK cells and binds to cadherins E, N, and R [58,59]. While N- and R- cadherins are widely expressed in the nervous system, E-cadherin is expressed on most epithelial cells [60]. However, E-cadherin is also found on peripheral blood cells and Langerhans cells, suggesting a broader range of cellular interactions along the KLRG1-E-cadherin axis [61]. In vitro experiments demonstrated, much in line with observations reported for NK cells, that KLRG1/E-cadherin engagement fosters inhibitory effects on human ILC2s by dampening their expansion, the expression levels of the transcription factor GATA3 and the release of cytokines [62] (Figure 2). Conclusively, downregulation of E-cadherin, a hallmark of the inflamed skin of atopic dermatitis patients revealed elevated IL-5 and IL-13 levels in dermal ILC2s and positions the KLRG1/E-cadherin interaction as new sensors of tissue damage [62].

#### 2.2.5. PD-1 (CD279)

Programmed cell death ligand 1 (PD-1) plays a well characterized role in modulating the exhaustion pathway in activated T cells. Ligands for PD-1, PD-L1, and PD-L2, are expressed by a variety hematopoietic and non-hematopoietic cells [63]. PD-1 expression was reported on ILC2s and NK cells where in the latter it has been shown to control NK cell functions [64,65]. In the former, PD-1 is important in the proliferation and cytokine production of both mouse and human ILC2s. In particular, Bcl11b-dependent PD-1 expression was reported for both ILC progenitors and ILC2s. Interestingly, IL-25R overexpression was sufficient to rescue the absence of PD-1 in Bcl11b-deficient ILCs [66]. Playing an inhibitory role on activated T cells, PD-1 was also found to negatively regulate KLRG1^+^ ILC2s through inhibition of STAT5 phosphorylation. As a consequence, ILC2 proliferation and cytokine production were significantly elevated in the absence of PD-1. As a result, blocking PD-1 enhanced ILC2-dependent anti-parasitic function during *Nippostrongylus brasiliensis* infection [65]. Interestingly, PD-L1 expression was reported on ILC2s following helminth infection and was found to boost Th2 cells, stimulating increased expression of GATA3 and production of IL-13 on these adaptive cells [67] (Figure 2). More recently, an IL-33-dependent upregulation of PD-1 on adipose tissue ILC2 conferred interactions with PD-L1^+^ adipose tissue macrophages and impaired tissue metabolism [68]. Strikingly, PD-1 blockade was able to partially restoring glucose tolerance and ILC2 functions to prevent obesity and sustain tissue homeostasis.

#### 2.2.6. NKp30 (NCR3/CD337)

The natural cytotoxicity receptors (NCRs) are an important family of activating receptors that include NKp30, NKp44, and NKp46. They function to initiate an immune reaction following recognition of cellular and viral ligands. Though most commonly used to identify NK cells, NCRs are also found to be expressed on specific ILC subsets [69]. These include NKp46 on ILC1s, NKp30 on ILC2s; and NKp30 and NKp46 on ILC3 subset [70]. In the context of ILC2s, NKp30 acts as an activating type I immunoglobulin-like transmembrane receptor in humans [71] (Figure 2A). Cytomegalovirus tegument protein pp65, Duffy binding-like—1α domain of *Plasmodium falciparum* erythrocyte membrane protein—1, nuclear factor HLA-B-associated transcript 3, and tumor associated cell surface protein B7-H6 have collectively been reported to engage NKp30 [72,73,74,75]. Recently, an NKp30-expressing ILC2 subset composing 52.4 ± 11.5% of all circulating human ILC2s were shown to bind B7-H6, to rapidly induce the production of type 2 cytokines, particularly IL-4, IL-5, IL-13, and GM-CSF as well as IL-2, IL-3, and IL-8 [76] (Figure 2). The authors further demonstrated NKp30-mediated activation downregulated the receptors for IL-25, IL-33 and prostaglandins on ILC2 to prevent feed-forward activation. In fact, the inflamed skin of atopic dermatitis patients was found to express elevated levels of B7-H6 compared to healthy controls and could thus be a possible ligand driver of NKp30-mediated ILC2-dependent type 2 immunity in these patients [76]. With approx. 50% of all circulating ILC2 in the blood expressing NKp30, Trabanelli et al. found that myeloid leukemia-derived PGD2 and surface expressed B7-H6 elevated ILC2-derived IL-13 production and synergized in creating an immunosuppressive milieu, fostering the development of monocytic myeloid-derived suppressor cells (M-MDSCs) [77]. Employing a humanized mouse model of myeloid leukemia, blockade of PGD2, IL-13, or NKp30 reversed ILC2-driven immunosuppression and increased the survival of leukemic mice [77]. Collectively, NKp30 ligation on ILC2 promotes type 2 immunity opening the road for therapeutic interventions, targeting NKp30 to ameliorate skin disease and facilitate anti-tumor immunity.

### 2.3. Lipid-Driven Modulation of ILC2

Bioactive lipids are able to modulate the activity of ILC2s as cell signaling messengers in both humans and mice. Leukotrienes (LTs), prostaglandins (PGs), and lipoxins (LXs) are bioactive lipids that regulate inflammation and lung homeostasis. These bioactive lipids are derived from arachidonic acid, an omega-6 polyunsaturated fatty acid that is primarily obtained from the diet [78]. Arachidonic acid is found attached to phospholipids in all mammalian membranes and is released by phospholipase A2 to generate lipid-derived mediators [79].

#### 2.3.1. Leukotrienes

The first step in the production of LTs through the 5-lipoxygenase pathway is the generation of LTA_4_ from arachidonic acid. LTA_4_ is rapidly converted to LTB_4_, or to the cysteinyl (Cys) LTs, LTC_4_, LTD_4_, and LTE_4_. CysLTs are highly active bronchoconstrictors and contribute to the pathogenesis of asthma and other allergic diseases as pro-inflammatory lipid mediators [80]. The G protein-coupled receptors, CysLT receptor 1 (CysLT1R) and CysLT2R, are the primary receptors binding to CysLTs, with the interaction ofLTD_4_ with this receptor being the strongest [81]. CysLT1R is expressed on the surface of airway smooth muscle cells, mast cells, eosinophils, dendritic cells, and macrophages and have recently been reported to be selectively expressed on Th2 cells [82,83,84,85]. Similar to Th2 cells, ILC2s are believed to contribute to type 2 lung inflammation through the secretion of IL-5 and IL-13. A role for LTs in regulating ILC2 activity was reported after identifying CysLT1R expression on mouse lung and bone marrow ILC2s [86,87]. ILC2s were further found to be activated by LTD_4_ with either IL-33 or LTD_4_ resulting in similar amounts of IL-4, IL-5, and IL-13 secretion, while very small amounts of cytokines were secreted with the addition of montelukast, a CysLT1R antagonist or in the absence of endogenous LT generation [86,87] (Figure 2).

#### 2.3.2. Prostaglandins

PGs are generated from arachidonic acid via the cyclooxygenase (COX) pathway. In this pathway, arachidonic acid is first oxidized into PGG_2_ by COX, followed by a reduction into PGH_2_ by peroxidase. PGH_2_ is an unstable intermediate that is quickly metabolized by specific PG synthases to form the five primary PGs, PGD_2_, PGE_2_, PGF_2_, PGI_2_, and thromboxane A_2_ [88,89]. As PGD_2_ and PGI_2_ are the only reported PGs known to regulate ILC2s, we will focus on their role in ILC2 biology. PGD_2_ is generated through the metabolism of PGH_2_ by PGD_2_ synthase in mast cells, eosinophils, and macrophages during allergic responses, and binds to the activating G-protein couple receptor CRTH2. As laid out earlier, CRTH2 is expressed on ILC2s, and Th2 cells has initially been used in humans to identify and distinguish ILC2s from ILC1s and ILC3s [90]. When PGD_2_ binds CRTH2, ILC2s are activated and execute their context-dependent role as discussed above (Figure 2A).

In contrast to PGD_2_, PGI_2_ inhibits ILC2 activation and has anti-inflammatory properties by reducing IL-5 and IL-13 secretion in ILC2 [91] (Figure 2A). PGI_2_ is synthesized through the metabolism of PGH_2_ by the prostacyclin synthase in endothelial cells, muscle cells, fibroblasts, follicular dendritic cells, and thymic nurse cells, and binds to the prostacyclin receptor (IP), which is expressed on Th1 cells, Th2 cells, bone-marrow derived DC and ILC2s [91]. Ex vivo stimulation of ILC2s with a PGI_2_ analog revealed an inhibitor effect on the IL-33-mediated secretion of IL-5 and IL-13 in bone-marrow derived ILC2s. PGI_2_-dependency of these inhibitory effects were confirmed to be IP specific using IP-deficient mice. Strikingly, PGI_2_-treatment abrogated even synergistic cytokine stimulation by IL-2 in combination with IL-33 making this PGI_2_, its synthesis pathway, and analogs, interesting therapeutic options for diseases driven by activated ILC2 [92].

#### 2.3.3. Lipoxins

Lipoxin A4 (LXA4) is a pro-resolving lipid mediator derived from arachidonic acid and is produced through the 15-lipooxygenase pathway. It is expressed in eosinophils, epithelial cells, and macrophages and binds to the ALX/formyl peptide receptor type 2 ALX/FPR2 that was recently found to be expressed on human NK cells and ILC2s. LXA4 inhibited the secretion of IL-13 by ILC2 stimulated with PGD_2_ or combinations of IL-25 and IL-33 (Figure 2). LXA4 stimulation further promoted NK cell-mediated apoptosis of eosinophils and decreased lung inflammation [93].

### 2.4. Regulation of ILC2 by Metabolites

Vitamin A, an important component of our diets, is converted in the bioactive metabolite retinoic acid (RA) via dendritic cells, macrophages, stroma cells, neurons, or the intestinal epithelium [94]. While it is an important metabolite for the transcriptional stability of the ILC3 lineage, RA engages RARs in ILC2 and impacts their activation and function [95] (Figure 2A). RA induces α4β7 integrin-dependent gut homing exclusively in humans ILC2s, while cytokine-mediated expansion and cytokine production by mouse ILC2s synergizes with RA [96]. Surprisingly, ILC2 were found to drive the production of RA by myeloid cells in the pancreas through IL-13 and GM-CSF (Figure 2B). Similar observations have been made for ILC3s in the intestinal tract [45,97]. ILC2-driven RA production by myeloid cells promoted Treg differentiation similarly to ILC3-driven RA production, but in turn regulated insulin production by beta-islet cells and glucose homeostasis. In the absence of vitamin A, ILC2s utilize dietary fatty acids to sustain their activity, in particular their production of IL-13 [98]. IL-13 production by ILC2 in turn impacts alternatively activated adipose tissue macrophages and the differentiation of adipocyte precursors [99]. Western diets are high in unsaturated lipids and the major cause of atherosclerotic plaques and cardiac complications. Ablation of ILC2s accelerated the development of these pathologies in mice and requires the lipid-mediated induction of IL-5 and IL-13 to ensure appropriate atherosclerotic protection [100]. Interestingly, short chain fatty acids (SCFA), the fermentation product of dietary fiber, play an opposing, inhibitory role on ILC2 function. Butyrate, one of the prominent SCFAs, suppressed cytokine production and proliferation of ILC2s likely independent of GPR41 or GPR43 and histone modifications [101] (Figure 2).

### 2.5. Neuronal and Hormonal Modulation of ILC2

Neuro-immune interactions initiate lymph node development via RA-driven CXCL13 production by stroma cells followed by the accumulation of CXCR5-expressing ILC3s [102]. These findings inspired the investigation of alternative, possibly direct pathways of neuro–ILC interactions. Indeed, tissue-resident ILC2 were reported to express beta2-adrenergic receptors (b2-AR), vasoactive intestinal polypeptide receptor 1 and 2 (VPAC1/2) and Neuromedin-U receptor 1 (NMUR1) [103,104,105,106,107] (Figure 2). While the expression of these receptors slightly differs on lung-resident ILC2, these receptors collectively allow ILC2 stimulation by sensory, sympathetic, and parasympathetic neurons [103,104,105,106,107]. Neuron-derived catecholamines engage b2-AR on ILC2s and suppresses their expansion and effector functions, contributing to the resolution of inflammation after parasitic infections [107]. The release of neuropeptides is a long-known communication axis between the nervous system and immune cells. The secretion of vasoactive intestinal peptide (VIP) by Nav1.8+ nociceptors encages VPAC1 and 2 receptors on ILC2s and increased the secretion of IL-5. This communication axis is thought to be important to sustain peripheral eosinophil numbers following circadian oscillation and metabolic stimulation [108]. Moreover, cholinergic neurons and the neuropeptide neuromedin U (NMU) were identified as potent stimulators of ILC2 functions in the lung and intestine. Engaging the NMU receptor 1 (NMUR1) drives the production of a diverse array of cytokines (IL-4, IL-5, IL-6, IL-10, and IL-13) by macrophages and eosinophils with implications in muscle contractility and allergic inflammation. Interestingly, NMU simulation of ILC2s alone results in IL-5 and IL-13 production through the calcineurin/NFAT and ERK1/2 pathways and yields cytokine production levels comparable to PMA/Iono or IL-2, IL-7, IL-25, and IL-33 stimulation. While ILC2 development is independent of the NMU-NMUR1 axis, its activation upon parasitic infection is important to lower parasite burden [103,104,105]. These findings collectively implicate the communication of neurons and ILC2s as elements of type 2 immune responses potentiating autoimmunity and antiparasitic responses.

### 2.6. Hormone-Mediated Sex Differences in ILC2 Function

Type 2 immune responses show a strong bias towards sex-differences across populations. For example, females are more prone to develop asthma than males. In line with these findings, data has demonstrated higher activation of ILC2s in asthmatic females when compared to their male counterparts [109,110]. These observations were then followed by reports demonstrating high expression levels of androgen receptors on ILC2s [111]. While female ILC2s produced higher levels of IL-5 and IL-13, cytokine production was blocked by dihydrotestosterone administration in females through direct regulation of RORa [112]. Collectively, these findings highlight the need to validate and control for ILC2-mediated sex differences in experimental disease models driven or ameliorated by ILC2 (Figure 2).

### 2.7. Control of ILC2 function by MicroRNA

MicroRNAs (miRNAs) are posttranscriptional repressors of gene expression. The role of miRNAs has been investigated in T cell-mediate type 2 immunity and was found to influence the development of asthma and allergy [113,114]. Specifically, miRNAs miR-24 and miR-27, were shown to co-operatively regulate Th2 cell differentiation through repression of the GATA3, Ikzf1, and IL-4 in mice [115]. Micro RNA 155 has been demonstrated to negatively regulate the production of IL-5 and IL-13, much in line with the observation that miR-155-deficient mice exhibit significantly reduced airway inflammation and eosinophilia in experimental models of allergic airway inflammation [116,117]. While initially described for Th2 cells, miR-155’s effects on type 2 immunity reproduced for ILC2. In line with an ameliorated allergic response, ILC2 numbers and their IL-13 production in response to IL-33 was impaired in *miR-155*^−/−^ mice [118]. However, the authors missed to address whether improvement of allergies in *miR-155*^−/−^ mice was the result of an ILC2-intrinsic defect of miR-155 or an effect of miR-155 depletion on the stromal or T-cell compartment. A more systemic analysis performed on naïve ILC2 or ILC2 isolated from IL-33 injected or helminth infected mice identified multiple immunoregulatory miRNAs that were upregulated following IL-33 administration or helminth infection, including miR-155 [119]. Mixed bone marrow chimera experiments confirmed an intrinsic role for miR-155 in preventing apoptosis in ILC2. Similarly to their robust activities within B and T cell development, the cluster miR-17~92 was identified to control ILC2 activity during allergy [120,121]. Similarly to miR-155, the miR-17~92 cluster promoted IL-5 and -13 production in response to IL-33 and ILC2-driven type-2 inflammation in vivo. These results suggest interconnected, but nonidentical pathways of gene regulation in ILC2s, independently targeted by distinct miRNAs. The particular importance of miR-155 in ILC2 survival may separate its physiological role from cluster miR-17~92. While it remains to be shown if other pathways of ILC2 stimulation use miRNAs as regulator, an overarching role for miRNAs in the regulation of type 2 immunity can be stated and further be explored.

## 3. ILC2-Tissue Crosstalk

ILC2s are present at diverse anatomical sites including barriers such as the lung, intestine, and skin where they have constant interaction with stromal and epithelial cells. ILC2s at these locations serve as rapid and innate source of cytokines to facilitate host defense and tissue repair [122,123]. Similar to other lymphocytes, ILC2 rely on cytokines for local survival signals, tissue-resident maintenance, and self-renewal, while activation occurs antigen-receptor independent in reliance on stimulation through TSLPR, T1/ST2, IL-17RB, and the IL-18R [124]. These receptors allow ILC2s to sense damaged or stressed tissue cells and facilitate their role as tissue-resident initiators of type-2 responses. IL-33, constitutively expressed and released after cell injury, requires proteolytic activation by mast cell- and neutrophil-derived proteases to initiate T1/ST2 receptor signaling on ILC2s, basophils, mast cells, and T_H_2 cells [125,126]. This mechanism can be observed via intranasal administration of allergens (such as *Alternaria* spp., papain, or ragweed). Elevated levels of bioactive IL-33 in the bronchoalveolar lavage (BAL) or nasal lavage fluids shortly after allergen exposure are rapidly detected due to the release of Chymase, Tryptase, Elastase, or Cathepsin G, suggesting an immune-mediated communication between tissue and ILC2 [127,128,129,130]. However, increase levels of *Il33* expression are found in patients suffering from chronic obstructive pulmonary disease (COPD), graft-versus-host disease (GVHD) and atopic dermatitis at sites of inflammation [131,132,133]. Similarly to humans, *Il33* expression is increased in alveolar type II pneumocytes following nematode infection, exposure to cigarette smoke, or intranasal challenge with allergens in mice [133,134,135,136,137]. In addition to epithelial and endothelial cells, activated fibroblasts, fibroblast-like cells, and myofibroblasts are important sources of IL-33 during inflammation, particularly in diseases or infections accompanied by tissue fibrosis, mucosal healing, and wound repair [138,139,140,141]. IL-33 plays a critical role in host defense to helminth infections via the cytokine-driven activation of ILC2, increased IgA levels and elevated goblet cell hyperplasia [123,135,137,142,143,144,145]. Although IL-33 confers beneficial host defenses, its role in the progression of auto-inflammatory disorders like asthma, rhinosinusitis, obesity, atopic dermatitis, and lung fibrosis are apparent. During allergic lung inflammation, IL-33 activated ILC2s are a potent innate source of type-2 cytokines that promote DC migration, IgE production, eosinophilia, goblet-cell hyperplasia, mucus overproduction, and smooth-muscle contraction [115,118,143,146,147,148,149,150] (Figure 2B). The localized actions of IL-33 on ILC2 activity were demonstrated in mice with skin-specific overexpression of IL-33 develop spontaneous dermatitis with increased ILC2 numbers; however, a conflicting report demonstrated that skin ILC2 activation is critically dependent on TSLP signaling [151,152]. While both cytokines activate ILC2s, they might not be the only tissue-derived form of ILC2 activation in the skin [62]. IL-18, also released by damaged tissues, was shown to selectively activate IL-18R-expressing skin-resident ILC2, a process contributing to the severity of atopic-dermatitis [25]. Interestingly, this suggests that IL-33 via ILC2 critically contributes to acute and chronic type-2 immunity across several tissues [153,154]. The activation of IL-33 or IL-18 are distinct and could reflect a rather tissue-specific mode of cell damage integrated into ILC2 activation. Interestingly, several reports suggested a critical crosstalk between ILC2s and Platelet Derived Growth Factor Receptor Alpha (PDGFRa) with the latter being an established player in the context of fibrosis, tissue repair, and cancer [155]. Two very elegant studies using IL-33 reporter mice identified PDGFRa-expressing fibroblasts in the lung and adipose tissue as a major steady state source of TSLP and IL-33. Adventitial stroma cells of the lung and adipose shared transcriptional similarity to mesothelial cells. These cells activated ILC2-dependent IL-13 release through TSLP. Production of IL-13 by ILC2 then triggered the release of IL-33 by these stroma cells, proposing an intimate ILC2-tissue cell circuit required for optimal type 2 immunity [156]. Interestingly, using a different IL-33 reporter strain, adipose stem and progenitor cells, and mesothelial cells were reported to contribute to ILC2 activation across all adipose tissues [157]. This establishes a connection between tissue-stroma and ILC2 in organ homeostasis and physiology.

IL-25 (IL-17E), the ligand for IL-17RB, is a member of the IL-17 family and is an important cytokine in ILC2/tissue crosstalk. Chemosensory tuft cells are the major source of IL-25 throughout the gastrointestinal tract, while chemosensory brush cells are the key source of IL-25 within the airway epithelium, even though alternative sources of IL-25 have previously been reported [158,159,160,161]. Similar to IL-33, IL-25 plays a major role in immunity to enteric parasitic infections, where intestinal IL-25 producing Tuft cells ‘taste’ microbial metabolites such as succinate, a metabolite secreted by protists and helminths. It is worth noting that other activating metabolites could also be released by helminths to triggers Tuft cell-specific IL-25 production [162,163,164]. IL-25 specifically activates IL-17RB-expressing inflammatory ILC2 (iILC2), capable of interorgan trafficking [165]. Inflammatory ILC2 migrate to the lung after injections of IL-25, or helminth infection, possibly following the worm throughout its lifecycle into other organs [26]. In line with the idea that several organs are affected by parasitic infections, a global induction of IL-25 secreting tuft cells following parasitic infection, emphasizes the role of IL-25-mediated activation of ILC2 across extra-intestinal locations. Being abundant in the gut, IL-25 has also been implicated in the pathogenesis of IBD. Interestingly, subsets of Crohn’s disease patients exhibit increased numbers of IL-13-producing ILC2s [166]. Additionally, oxazolone-mediated experimental colitis was driven by IL-25-dependent activation of ILC2s [167]. Collectively, these reports support a possible role for IL-25-mediated type 2 immunity in the pathogenesis of intestinal auto-inflammation. In summary, ILC2s form a close relationship with non-hematopoietic tissue cells and serve as first responders to alarmins released into the tissue microenvironment. This crosstalk is vital during host defense against parasitic infections and tissue-repair, but propagates autoimmunity, fibrosis, and cancer under uncontrolled circumstances.

## 4. Protozoan Commensals as Regulator of ILC2 Activity

The intestinal microbiome is composed of several microbial kingdoms. Archaea, viruses, bacteria, worms, fungi, and protozoa have been found in the intestinal tracts of otherwise healthy humans. Interestingly, all of these kingdoms were demonstrated to transform the local and peripheral immune environment while peacefully coexisting [168]. However, several members of the above listed kingdoms are considered pathobionts due to their potential to cause pathologies under genetic or environmental perturbations of the host [169]. Even though colonization by pathobionts is a stable process, particularly for bacteria, helminths and protozoan colonization are often transient, resulting in the elimination of the microbe from the intestinal tract [170,171,172].

Less aggressive protozoan species have been identified in healthy individuals, without causing any obvious signs of inflammation or disease [173]. *Dientamoeba fragilis* is one prominent species, closely related to enteric *Trichomonads*, which are frequently found in the intestinal tract of humans [173,174]. Enteric protozoan species and particularly their interactions with gut-resident immune cells are far less well understood. Considering their abundance in healthy individuals, their implication in mucosal immunology is an intriguing area of biology. The identification of *Tritrichomonas* species as commensal microbes in healthy animals across many non-commercial animal facilities inspired research to identify their importance in the host–microbe crosstalk. *Tritrichomonas muris* (*T.muris*) and *Tritrichomonas musculis* (*T.mu*) were reported to permanently colonize the intestinal tract of mice, persist in these species and permanently colonize their offspring. Even though colonization lasts for the entire life of the animals, little to no effect is reported concerning the health status of mice carrying a protozoan commensal [173,175]. Interestingly, colonization of mice with *T.muris* or *T.mu* biased the threshold of the colonic intestinal immune activation towards Th1 and Th17 polarization, increasing the susceptibility of mice to develop inflammatory bowel disease in the T cell transfer model of colitis [173,175]. The elevated bias towards Th1 activation within the large intestine further enabled *T.mu*-colonized mice to develop a resistance to infections by enteric pathogens like Salmonella [172].

**Figure 3:**
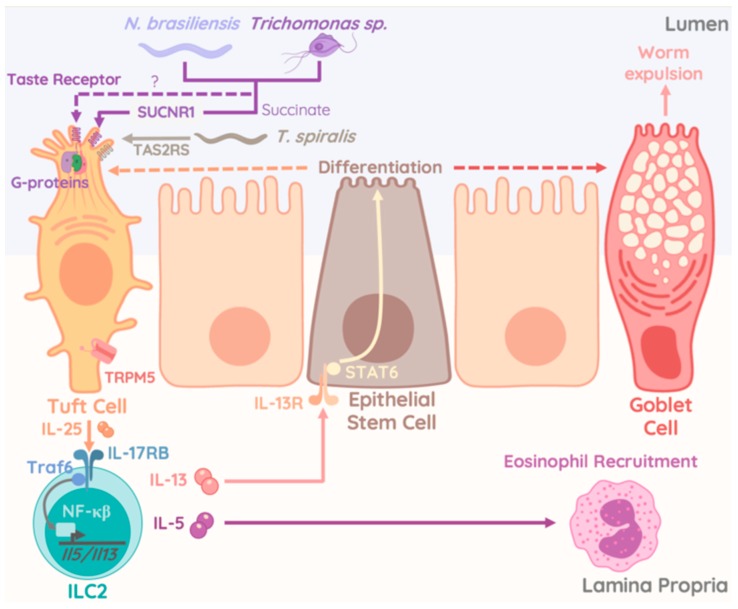
Interactions of worms and protozoa with epithelial cells regulates ILC2-driven type 2 immunity. Picture elucidates the pathways of ILC2 activation upon recognition of parasitic helminths or commensal protozoa. The sensory and regulatory functions within the intestinal epithelium are represented by an array of taste receptors expressed on Tuft cells (including TAS2RS, a *T. spiralis* specific receptor). Engagement of these receptors triggers the activation of G-proteins and synergizes with the succinate-triggered succinate receptor (SCNR1). As a result, Tuft cells release IL-25 that in turn engages the IL-25 receptor (IL-17RB) on ILC2. Activation of TRAF6 and NFkB downstream of IL-17RB initiate the production of IL-5 and IL-13. These cytokines, released by ILC2 mediate the recruitment of eosinophils through IL-5 and the activation of epithelial stem cells through IL-13. IL-13 mediated activation of STAT6 then drives differentiation of epithelial stem cells into goblet cells and tuft cells, which in turn facilitate worm expulsion and anti-helminth immunity.

However, analysis of the small intestinal tract revealed additional significant changes following *Tritrichomonas* colonization. An increase in goblet cell hyperplasia, epithelial proliferation, and cell death, as well as elevated numbers of DCLK1-expressing Tuft cells were reported in mice colonized with *T.muris* and *T.mu* [172,176] (Figure 3). Goblet cells expansion, a consequence of ILC2-derived IL-13 stimulation was initiated through taste-receptors, requiring cation channel TRPM5 in taste signal transduction, on Tuft cells upon sensing of *Tritrichomonas* species and enteric helminths [158,159,176] (Figure 3). Interestingly, ILC2 numbers and cytokine production were increased in mice colonized with *Tritrichomonas* species and unleashed Tuft cell differentiation via the IL-13-STAT6 pathway. Similar findings were made in mice deficient in the E3-Ubiquitin ligase A20. Selective deficiency of A20 in ILC2 resulted in elevated IL-13 production and ILC2 proliferation. In turn, higher levels of tuft cells were observed alongside a significant elongation of the entire small intestinal tract. Dietary metabolites like acetone or succinate, released by the microbiome were found to engage the Succinate receptor 1 (GPR91) on Tuft cells and were found to be a potent trigger of ILC2 cytokine production and proliferation upstream of the A20-mediated inhibition of ILC2 [164]. A similar report demonstrated that *Tritrichomonas rainier*, a related species to *T.mu* and *T.muris*, initiated GPR91-dependent Tuft cell-ILC2 communication via IL-25 [163]. Strikingly, the authors provided evidence that succinate-mediated activation of Tuft cells by protozoan-derived succinate triggered a unique activation pathway for the Tuft cell-ILC2 crosstalk that was dispensable for appropriate anti-helminth response [164]. A recent study identified the TAS2R family of taste receptors on Tuft cells as specific signaling unit that mediated the release of IL-25 when engaged by *Trichinella* secreted compounds. This mechanism required the interactions with TRPM5 and was counterbalanced by inhibitors of G-protein activity and the inositol triphosphate receptor type 2. While TAS2Rs showed specific upregulation upon stimulation with *Trichinella spiralis*-derived compounds, these data sets collectively suggest a broad sensory potential by epithelial tuft cells [177] (Figure 3). Specific reactivity to chemicals produced by specific kingdoms of enteric microbes including bacteria, protozoa, or helminths could activate the Tuft cell-ILC2 circuit in a taste-receptor specific fashion and trigger host immune adaptations or disease exacerbation in the context of specific intestinal microbes.

The underappreciated prevalence of *T. mu* orthologs that colonize the human gastrointestinal tract, such as *Pentatrichomonas hominis* and *Dientamoeba fragilis*, could have major implications in the development and severity of human disease. The expansion and release of iILC2 from the intestine into the circulation in an S1P dependent manner critically requires IL-25 [26,165]. A protozoan-stimulated Tuft cell-ILC2 circuit propagated by IL-25 could therefore be a mechanism promoting systemic dissemination of ILC2. The long-term consequence of these newly distributed resident ILC2 in tissues is not known, but could be of persistent nature considering that activated iILC2 retain an epigenetically-poised memory that would allow them to rapidly and more robustly respond to re-challenge [178]. While gut-derived ILC2 do exhibit some plasticity and could revert to a non-inflammatory state over time, commensal protozoan colonization was found to last for the lifetime of the host and therefore could continually seed distal tissues with increasing numbers of ILC2 for years [26,165]. Although protozoan colonization can be asymptomatic or be accompanied with minor enteric complications [173,174,175], it is not known if colonization may also be a contributing factor to more chronic conditions and could therefore be a risk factor for autoimmune disorders such as IBD. The microbiome is already a well-established regulator of the mammalian immune system with disruption of microbial composition in the intestine possessing long reaching effects on immunity at distal sites. Therefore, a better understanding of the role of protozoan commensals in autoimmune disorders and as modulators of tissue immunity could inspire new specific anti-microbial therapies that could be a future treatment for certain inflammatory disease. Multiple species of *Tritrichomonas* spp. have been discovered and reported in quick succession [163,173,175], with little to no knowledge on their actual phylogenetic relationships. This highlights a gap in our understanding of the diversity of the protozoan microbiome in healthy mice and humans. Future studies should elucidate the full diversity of human commensal protozoa and explore the effects that colonization with different species could have on immune modulation by tissue-resident ILC2 within the intestine and beyond. Conversely, given that S1P inhibitors are in trials for treatment of multiple sclerosis and inflammatory bowel disease, similar drugs could potentially be used to block S1P-dependent ILC2 trafficking and help treat patients that suffer from chronic type 2 autoimmunity like asthma or dermatitis [179,180]. As we gain more insight into the symbiotic relationship between commensal protists and our immune system more targeted therapeutics to modulate ILC2 function for the improvement of inflammatory conditions will emerge.

In essence, ILC2s pose an important element of the body-wide type 2 immunity. The diversity in their development, functional specialization, and tissue localization make these cells a highly interesting innate immune cell type. Their unique involvement in host–pathogen/host–microbiome interactions renders these cells an important possible target for the therapeutic improvement of host defense. However, their dynamics and long-term adaptations, as well as contextual contributions require further research to understand the possible impact of their manipulations during tissue and immune homeostasis in settings of dysregulation and auto-inflammatory type 2 immunity. In particular, questions and areas of research interest are:

1. Are there several pools of ILC2 precursors?
2. What is the contribution of postnatal and adult ILC2 to tissue homeostasis and host defense?
3. Which are ILC2 specific tissue niches?
4. What is the role of Tuft cell subsets across tissues in regulating ILC2 activity?
5. Exploring the potential of ILC2s in immunotherapy during host immunity.
6. Identifying lineage determining transcription factors in human ILC2 for the use in iPSC technology.
7. Developing ILC2-specific Cre/reporter lines for faithful tracking of ILC2 subset or ILC2 at distinct developmental stages.

Lastly, with enteric protozoa being a new element that regulates ILC2 activity, investigations into the intestinal ‘Eukaryome’ of mice and humans will be mandatory to inform about the diversity of intestinal protozoa and their unique role in exacerbated gut type 2 immunity. This area of research faces several challenges. First, isolation and culturing protocols for most enteric protozoan commensal do not exist or require complicated co-cultivation with commensal bacteria. Genome and transcriptome data for intestinal commensal protozoa have never been generated. Assembly of protozoan transcriptome data will be particularly challenging due to the extreme size and the large abundance of repetitive element within protozoan genomes. Similar to current microbiome research in bacteria, analysis, and data set generation for new protozoan commensal will inspire a new area of research possibly able to identify new mechanisms of host–microbiome interactions at mucosal surfaces with a target set on type 2 immunity.
